# Human Adipose-Stem-Cell-Derived Small Extracellular Vesicles Modulate Behavior and Glial Cells in Young and Aged Mice Following TBI

**DOI:** 10.3390/cells14171304

**Published:** 2025-08-22

**Authors:** Salma S. Abdelmaboud, Lauren D. Moss, Charles Hudson, Rekha Patel, Marta Avlas, Jessica Wohlfahrt, Tiara Wolf, Jennifer Guergues, Stanley M. Stevens, Niketa A. Patel, Paula C. Bickford

**Affiliations:** 1 Department of Molecular Pharmacology and Physiology, University of South Florida Morsani College of Medicine, Tampa, FL 33612, USA; salma4@usf.edu; 2 Department of Neurosurgery, Brain and Spine, University of South Florida Morsani College of Medicine, Tampa, FL 33612, USA; ledaly@usf.edu (L.D.M.); avlas@wisc.edu (M.A.); 3 James A. Haley Veterans Hospital, Research Service, Tampa, FL 33637, USA; chudson1@usf.edu (C.H.); rekha.patel1@va.gov (R.P.); niketa.patel@va.gov (N.A.P.); 4 Department of Molecular Biosciences, University of South Florida, Tampa, FL 33620, USA; jwohlfahrt@usf.edu (J.W.); jguergues@usf.edu (J.G.); smstevens@usf.edu (S.M.S.J.); 5 Department of Molecular Medicine, University of South Florida Morsani College of Medicine, Tampa, FL 33612, USA

**Keywords:** TBI, aged, small extracellular vesicles (sEVs), human adipose derived stem cells (hASC), microglia, astrocytes, neuroinflammation

## Abstract

Traumatic brain injury (TBI) is a major cause of long-term neurological impairment, with aging amplifying vulnerability and worsening recovery. Older individuals face greater cognitive and motor deficits post-TBI and respond less effectively to treatments, as both aging and TBI independently elevate neuroinflammation and cognitive decline. This study evaluated the therapeutic effects of human adipose-derived stem cell small extracellular vesicles (hASC-sEVs) on neurological recovery and neuroinflammation in a mouse model of TBI. Male C57BL/6 mice (3, 15, and 20 months old) underwent controlled cortical impact (CCI) and received intranasal hASC-sEVs 48 h post-injury; control groups received PBS. A dose–response study at 7 days post injury (dpi) identified 20 µg as the optimal therapeutic dose, improving motor function, reducing neuroinflammation, and enhancing neurogenesis. This was followed by a 30-dpi study assessing cognitive function, neuroinflammation, neurogenesis, and proteomic changes in microglia and astrocytes via mass spectrometry. hASC-sEV treatment significantly improved behavioral outcomes and reduced neuroinflammatory markers (GFAP, IBA-1, and MHC-II), with reduced efficacy observed in older mice. Proteomics revealed that hASC-sEVs reduce inflammatory proteins (TNF-α, IL-1β, IFNG, CCL2) and modulated mitochondrial dysfunction and reactive oxygen species. These results highlight hASC-sEVs as a promising cell-free therapy for improving TBI outcomes, especially in aging populations.

## 1. Introduction

Traumatic brain injury (TBI) is a global medical challenge and leading cause of a variety of disorders, including chronic memory deficits, dementia, tau pathology, psychiatric illness, epilepsy, stroke, and neurodegenerative diseases [1]. The worldwide incidence of TBI is estimated at 69 million cases annually [2]. In the United States (US), the Center for Disease Control and Prevention (CDC) has reported that about 1.5 million Americans survive a TBI each year, with around 230,000 requiring hospitalization [3]. According to the most recent data from CDC, 2025, individuals aged 75 and older had the highest numbers and rates of hospitalizations and deaths related to TBI. This age group represents about 32% of TBI-related hospitalizations and 28% of TBI-related deaths. TBI pathophysiology consists of sequential events that begins with a primary response to the acute phase of injury and progresses over time to a secondary response involving neuroinflammation that can result in neurological dysfunction. At the cellular level, secondary injury response is mediated by several pathways, including, but not limited to, (1) an excess of extracellular glutamate neurotransmitter causing excitotoxicity [4], (2) free radicals release that lead to neuronal cell damage [5], and (3) the neuroinflammatory response, which leads to a long-term TBI outcomes mediated by immune activation [6].

Age at the time of injury is a well-known independent predictor of worse outcomes following TBI [7] as aging is associated with increased morbidity and mortality from acute and chronic brain injuries compared to younger ages [8]. Aged people are vulnerable to more complicated neurological deficits and impaired functional recovery after TBI [9]. The effects of aging are highly evident in the immune system, as the development of an “inflammaging”, defined as a chronic, low-grade sterile inflammation, contributes to cognitive decline. This condition worsens when coupled with TBI, further exacerbating cognitive impairment [10]. Cellular senescence, along with the formation of a proinflammatory phenotype recognized as a senescence-associated secretory phenotype (SASP), enhances inflammaging and cognitive aging [11]. The age-related changes in immune response have been proposed to play a role in the poorer neurological outcomes observed in aged TBI animals since SASP-associated chronic inflammation in aged brains creates an environment that promotes disease and dysfunction post-TBI [12]. This is supported by research showing that, following TBI, aged animals demonstrated heightened signs of increased lipid peroxidation markers indicating oxidative stress (e.g., 4-hydroxynonenal, acrolein), along with a reduced capacity of antioxidant enzymes such as urate and ascorbate when compared to younger animals [13,14]. In addition, prolonged activation of microglia and astrocytes in the hippocampus associated with posttraumatic neuroinflammation is also profoundly affected by aging [15].

Microglia are the primary immune cells of the central nervous system (CNS), playing crucial roles beyond immune surveillance, including regulation of neurogenesis, synaptic pruning, and neurotrophin production during injury. In young brains, microglia maintain a balanced response to injury, aiding neuroprotection [16,17]. However, in the aged brain or post-traumatic brain injury (TBI), microglia often exhibit a primed, proinflammatory phenotype characterized by increased expression of markers such as MHC II, IL-1β, TNF-α, CD86, and ED1, along with a reduced response to anti-inflammatory signals like IL-10 and IL-4 [18,19,20,21]. Following TBI, microglia transition from a ramified to hypertrophic or bushy morphology and upregulate Iba-1, reflecting changes in activation state. These changes are more pronounced in aged TBI brains, with reduced ramified microglia and elevated reactive forms in regions such as the cortex, dentate gyrus, and thalamus [22,23].

Astrocytes are another key glial population in the CNS, supporting neuronal and vascular function through calcium signaling, regulation of blood flow, and release of vasoactive molecules [24,25]. Post-TBI, astrocytes undergo astrogliosis, marked by hypertrophy, GFAP and vimentin upregulation, and glial scar formation [26]. While the scar serves to isolate damaged tissue and restore the blood–brain barrier, it may also hinder axonal regeneration. Astrocyte reactivity impairs glutamate clearance, exacerbating excitotoxicity [26,27,28].

Microglia and astrocytes engage in dynamic crosstalk following TBI, mediating both protective and deleterious responses through bidirectional signaling. Microglia respond to astrocytic ATP release and DAMPs via purinergic receptors (P2X4, P2Y12), clearing debris and coordinating with infiltrating immune cells. However, excessive activation propagates neuroinflammation via reactive oxygen species and purines, contributing to secondary injury and neurocognitive decline [29,30,31,32].

Despite extensive preclinical research, translating neuroprotective strategies for traumatic brain injury (TBI) into effective clinical therapies remains a major challenge [33]. Among emerging approaches, cell-based therapies have shown considerable promise [34]. Adipose tissue offers an abundant and easily accessible source of mesenchymal stem cells (MSCs) with robust regenerative and differentiation capacities, comparable to MSCs from other tissues and unaffected by donor age [35]. Human adipose-derived stem cells (hASCs), in particular, offer advantages such as high isolation efficiency and minimal ethical concerns compared to embryonic stem cells, making them attractive candidates for regenerative applications [36].

hASCs secrete a broad spectrum of bioactive molecules that modulate the local tissue microenvironment and contribute to immunomodulation, neurite outgrowth, angiogenesis, and tissue repair. A significant portion of these therapeutic effects is mediated by extracellular vesicles (EVs), particularly small extracellular vesicles (sEVs), which carry proteins, mRNAs, and noncoding RNAs. These vesicles retain the therapeutic functions of their parent cells, including neuroregeneration, anti-apoptotic activity, oxidative stress reduction, and tissue remodeling. Due to their cargo composition and ability to evade immune rejection, sEVs have gained recognition as promising therapeutic agents [37,38,39].

Growing evidence supports the potential of MSC-derived sEVs in promoting functional recovery following TBI [40,41,42,43]. They modulate immune responses, suppress neuroinflammation, and support repair through their bioactive cargo. Our previous work demonstrated that intravenous administration of hASCs improved motor and cognitive outcomes in young rats post-TBI but was less effective in aged animals [44]. We also found that intranasal delivery of hASC-sEVs at 48 h post-injury enhanced recovery in young mice [45,46]. Nonetheless, their therapeutic efficacy in aged TBI models remains to be established. In addition to their neuroprotective roles, MSC-EVs promote neuronal and astrocytic differentiation, modulate T cell responses, and mitigate oxidative stress [47,48,49,50,51,52,53]. While promising, further investigation is needed to optimize delivery strategies, dosing, and therapeutic timing to fully realize the clinical potential of MSC-derived EVs, particularly in aging populations.

## 2. Materials and Methods

### 2.1. Animal Care

All surgical procedures were conducted in accordance with the University of South Florida Institutional Animal Care and Use Committee (IACUC) guidelines, with efforts made to minimize animal discomfort. Male C57BL/6 mice of three age groups, young (3 months), middle-aged (15 months), and aged (20 months), were used. Mice were housed in the USF Comparative Medicine (COM) facility under standard conditions (20 °C, 50% relative humidity, 12-h light/dark cycle) for one week before experimentation. Animals were randomly assigned to one of three groups: Sham, TBI + PBS (untreated), or TBI + sEVs (treated). Traumatic brain injury was induced via controlled cortical impact (CCI), while sham animals underwent only a midline scalp incision. All experiments were performed by investigators blinded to treatment conditions.

### 2.2. Controlled Cortical Impact (CCI) Mouse Model

Young (3-month-old) and aged (15- and 20-month-old) male C57Bl/6 mice were anesthetized with 1–2% isoflurane in a mixture of 60% nitrous oxide and 30% oxygen and secured in a stereotaxic frame (David Kopf Instruments, Tujunga, CA, USA). Anesthetic depth was confirmed via respiration rate and pedal reflexes. Body temperature was maintained at 37 °C using a heating pad. After applying ophthalmic ointment, a midline scalp incision was made to expose the skull. A 4 mm craniectomy was performed on the left lateral skull at coordinates 0.2 mm anterior–posterior and 0.2 mm medial–lateral from bregma using a handheld drill, and the bone flap was removed. CCI was induced using a 3 mm impactor tip angled at 20° to match cortical curvature, with parameters set to 4.0 m/s velocity, −0.95 mm depth (mild CCI), and 300 ms dwell time. The cranial window was left uncovered to avoid exacerbating cerebral edema and increased tissue damage associated with immediate skull reconstruction [54,55]. Incisions were sutured post-impact, and animals were placed in a heated recovery chamber until ambulatory. Postoperative monitoring was conducted, and all animals were maintained on a standard rodent diet. Sham controls underwent the same procedure, excluding craniectomy and cortical impact.

### 2.3. Small Extracellular Vesicle (sEV) Isolation and Collection from Human Adipose Stem Cells (hASCs)

#### 2.3.1. Cell Culture

The human adipose stem cells (hASCs, ZenBio, Durham, NC, USA #ASC-F-SL) were pooled from lean female subcutaneous depot with an average BMI of 27.9. All donors were non-smokers and non-diabetic. For the collection of conditioned media (CM), hASCs were grown to 90% confluence (8 × 10^6^ hASCs) in T75 flasks, and then the cell medium was replaced with serum-free mesenchymal stem cell basal media, with StemFlex medium kit (Gibco LifeTechnologies, Waltham, MA, USA, # A33494-01). The CM derived from hASCs was collected after 48 h.

#### 2.3.2. sEVs Isolation and Characterization

The CM (as described in Section 2.3.1) was then used to isolate small extracellular vesicles (sEVs). CM was centrifuged at 3000× *g* for 15 min to remove any hASC cell debris. The CM was concentrated using a 10 kDa molecular weight cut-off filter (MWCO). The CM was then processed to isolate EVs as described previously [56]. EVs were isolated using qEV columns (35 nm from IZON, Medford MA, USA). The qEV columns were prepared following the manufacturer’s instructions using PBS as a buffer. The qEV column was mounted on the Automated Fraction Collector (AFC, from IZON), which enables the reproducible and exact collection of fractions by size exclusion chromatography. The concentrated CM was then added to the prepared qEV columns. The sEVs were eluted in PBS, and fractions were collected every 500 μL. The sEVs were characterized as previously established by our lab [56,57,58]. Western blot was used to probe for tetraspanins CD9, CD63, and CD81 for verifying sEVs [58]. Nanoparticle tracking analysis (NTA3.1, Build 3.1.46 RRID SCR-014239) was used to analyze size and concentration of sEVs in each fraction (Figure S1A). The fractions containing a single peak of sEVs particles within the average particle size range of 90–275 nm were used in the experimental setups [56]. Automated Western blotting was performed as previously described [57] using the ProteinSimple JESS system (ProteinSimple, Santa Clara, CA, USA) according to the manufacturer’s protocol. Samples were separated using 12–230 kDa capillary separation cartridges, and 1 mg/mL of protein was loaded per run. Immunodetection was carried out using antibodies against CD9, CD63, TSG101, and ALIX to confirm the presence of canonical sEV markers (Figure S1B–D).

### 2.4. Intranasal Delivery of sEVs

At 48 h post-CCI surgery, mice were lightly anesthetized with 1–2% isoflurane in a gas mixture of 30% nitrous oxide and 60% oxygen. Small extracellular vesicles (sEVs) were administered intranasally using a P10 pipette tip, with equal volumes delivered bilaterally to each nostril. Control groups (sham and untreated TBI) received phosphate-buffered saline (PBS), while treatment groups received sEV. The administered volume was based on the concentration of sEV to achieve a total dose of 10, 20, or 50 µg per mouse, with no more than 5 μL applied per nostril per dose. A recovery period of 10 min was allowed between nostril administrations. We tested doses of 10 and 20 µg in 3- and 15-month-old mice. However, anticipating a reduced therapeutic response in 20-month-old mice, we additionally evaluated a higher dose of 50 µg alongside 10 and 20 µg.

### 2.5. Behavioral Tests

#### 2.5.1. Elevated Body Swing Test (EBST)

The Elevated Body Swing Test (EBST) assesses motor asymmetry commonly observed following unilateral brain injury [59]. In this test, mice are placed in a Plexiglas enclosure and habituated for 2 min. Each mouse is then lifted by the tail approximately one inch above the surface, allowing swings greater than 10° from the vertical axis to be recorded as left or right swings. Twenty swings are recorded per animal. A score of 10 indicates no motor bias, while scores ≥14 reflect significant asymmetry and motor deficits. The number of swings made to the higher side is averaged across treatment groups. Baseline assessments were conducted one day prior to controlled cortical impact (CCI) to confirm no pre-existing bias.

At 24 h post-CCI, mice were re-evaluated and randomly assigned to three treatment groups (sham, TBI + PBS, and TBI + 20 µg EVs) with comparable baseline EBST scores. Behavioral assessments were then conducted on days 1, 3, and 5 post-CCI. EBST was conducted in staggered cohorts post-CCI, with data pooled across both experimental endpoints, resulting in large N’s for this test only.

#### 2.5.2. Y Maze Test

The Y-maze test was used to evaluate spatial working memory and hippocampal function following TBI in mice. The apparatus consisted of three identical arms (A, B, and C) arranged at 120° angles. Each mouse was introduced into the starting arm (A), facing away from the center, and allowed free exploration for 10 min. Movements were recorded using an overhead video tracking system and analyzed with Anymaze software, V7.01. Successful working memory performance was defined as spontaneous alternation behavior, where the mouse entered all three arms in succession (a correct triad), avoiding re-entry into the most recently visited arm. Repeated entries into the same arm were counted as errors. The percentage of correct triads and the number of repeated arm entries were manually scored by an observer blinded to treatment groups. The maze was cleaned with 70% ethanol between tests to eliminate olfactory cues.

#### 2.5.3. Open Field Test (OFT)

The open field test measures exploration and anxiety-like behavior [60]. Briefly, a mouse was placed into an open field chamber square box that measured 40 × 40 cm under moderate lighting. The mouse was allowed to freely explore the 2 adjacently located imaginary square zones (center and perimeter) for 10 min. The open field box was thoroughly cleansed using 70% ethanol between sessions to preclude odor recognition. The exploration tracks were recorded with a video camera, and distance traveled and average speed were recorded and analyzed by the ANY-maze behavioral tracking system (Stoelting Co., Wood Dale, IL, USA). We tested mice on an open field 24 h prior to the novel place/novel object recognition test as a habituation phase.

#### 2.5.4. Novel Place/Novel Object Recognition Test (NPNOR)

This test was used to assess spatial memory and recognition in mice. The day following open field testing, the mice were placed back into the 40 × 40 square chamber and allowed to explore two identical objects (familiar objects) that were placed equidistantly apart and from the center for 5 min to familiarize the mice with the objects. The mice were removed from the chamber and placed in a holding area for 5 min. The mice were then returned to the testing area to allow for another 5 min of interaction with the familiar objects. This was repeated for a total of three training trials. Mice were then transferred to their home cage. At 4 h after the familiarization phase, novel place testing was performed, which involved moving one of the familiar objects to the opposite corner of the chamber, and the interaction of the mice with the objects was recorded for 10 min. The mouse was removed and placed in a holding area for 3 h. Then novel object testing was performed, which substituted one of the familiar objects with a new object, and exploratory behavior was again recorded for 10 min. All objects were thoroughly cleansed using 70% ethanol between sessions to preclude odor recognition. The exploration tracks were recorded with a video camera using ANY-maze software, V7.01. The investigation times for the objects were hand-scored by an individual blinded to the treatment groups. Score investigation was carried out when the mouse head was oriented towards the object and within 10 mm of the object perimeter.

### 2.6. Immunohistochemical Analysis (IHC)

#### 2.6.1. Brain Collection and Sectioning

Mice were sacrificed in a CO_2_ euthanasia chamber followed by transcardial perfusion with ice-cold 0.1 M phosphate-buffered saline (PBS) at pH 7.2 to remove blood from the vasculature. The brains were drop-fixed into 4% paraformaldehyde (PFA) in 0.1 M PBS at pH 7.2 overnight. They were then transferred to 30% sucrose in PBS for 24 h and allowed to sink. Brains were sectioned coronally at a thickness of 40 μm using a cryostat, and sections were collected in 96-well plates and stored in a cryoprotectant solution in a −20 °C freezer until further processing. Sections were used in both IHC and lesion volume analysis.

#### 2.6.2. Microglia, Astrocytes, and Neuron Marker Staining and Measurement

Immunostaining was conducted on every sixth coronal section encompassing the TBI impact region. Free-floating sections were first incubated in a solution containing 30% methanol and 3% hydrogen peroxide in PBS to quench endogenous peroxidase activity. After rinsing with PBS, tissues were incubated in a blocking buffer composed of 10% goat serum and 0.3% Triton X-100 in PBS for one hour to reduce nonspecific binding. Tissues were then incubated in PBS-TS solution (3% goat serum/0.1% Trition X-100/PBS) with primary antibody of anti-GFAP (1:10,000, # Z0334, DAKO, Santa Clara, CA, USA), anti-Iba-1 (1:2000, #019-19741, Fujifilm WAKO, Richmond, VA, USA), anti-major histocompatibility complex (MHC class II) (1:2000, # 55999, BD Pharmingen) or anti-doublecortin (DCX) (1:5000, #4604S, Cell Signaling, Danvers, Massachusetts, USA) overnight at 4 °C on a shaker (60 rpm). The following day, sections were washed in PBS-TS and then incubated with goat anti-rabbit secondary antibody for GFAP (1:500), IBA-1 (1:500), doublecortin (1:1000), and goat anti-rat secondary antibody for MHC-II (1:5000) for one hour at room temperature. Avidin–biotin substrate complex (Vector Laboratories, Newark, CA, USA) was used to amplify the signal for one hour. Tissue sections were developed using 3, 3′-Diaminobenzidine tetra-hydrochloride (Sigma, St. Louis, MO, USA) for GFAP and doublecortin or metal enhanced with cobalt chloride (Sigma, St. Louis, MO, USA) for IBA-1 and MHC-II. Free-floating sections were mounted onto glass slides and air-dried overnight. The following day, slides were dehydrated and cover-slipped using DPX mounting medium. Stained tissues with GFAP, IBA-1, and MHC-II were scanned using a Zeiss Axio Scan (ZEISS Microscopy, Munich, Germany) and quantitative analysis was conducted with NearCYTE image analysis software (http://nearcyte.org). Regions of interest were delineated on each section, and the area of positive staining was measured. DCX-stained tissues were visualized under a Nikon E600 light microscope and quantified using unbiased stereology with Stereo Investigator software, V1.3 (MicroBrightField Inc., Williston, VT, USA).

#### 2.6.3. Lesion Volume Analysis

For lesion volume assessment, every sixth coronal brain section encompassing the entire TBI-affected region was analyzed. Sections were mounted on glass slides, air-dried, and stained with hematoxylin QS (Vector Laboratories, catalog # H-3404) for 5–10 min, followed by a 5 min rinse in tap water. Slides were then left to dry overnight, dehydrated, and cover-slipped using DPX mounting medium. Lesion volume was quantified by visualizing stained sections under a Nikon E600 light microscope, Melville, NY, USA. The injured area in the ipsilateral hemisphere was delineated and measured using the Cavalieri estimator method with Stereo Investigator software (MicroBrightField Inc.).

### 2.7. Microglia and Astrocytes Isolation

Mice were sacrificed in a CO_2_ euthanasia chamber followed by transcardial perfusion with ice-cold saline to remove blood from the vasculature. Mice were then decapitated, and brains were collected. Each brain was dissected along the midsagittal plane, contralateral and ipsilateral to the brain lesion, and each half was weighed. The brains were dissociated using the Adult Brain Isolation Kit (Miltenyi Biotec, Bergisch Gladbach, GR, Germany). The half-brain was sliced into 4 sagittal pieces and then placed in a gentleMACS C-tube (Miltenyi Biotec, Bergisch Gladbach, GR, Germany) containing an enzymatic digestion mix provided in the kit. The C-tube was attached to the gentleMACS Octo dissociator with heaters (Miltenyi Biotec, Bergisch Gladbach, GR, Germany) and the kit program was run. The brain homogenate was centrifuged briefly at 300× *g*, resuspended, and strained using a 70 μm cell strainer. The samples were centrifuged again at 300× *g* for 10 min at 4 °C, and the supernatant was aspirated. Myelin was removed and red blood cells lysed using kit-provided reagents. Finally, the cell suspension was stained with mouse Fc receptor block (# 130092575, Miltenyi Biotec) at 2–8 °C in the dark for 10 min, followed by fluorophore-conjugated CD11b (#101208, Biolegand, San Diego, CA, USA), CD45 (#103114, Biolegand, San Diego, CA, USA), and ACSA-2 antibody (#130117386, Miltenyi Biotec) at 2–8 °C in the dark for 30 min. Microglia and astrocytes were sorted on a FACSMelody cell sorter into microcentrifuge tubes. They were identified as CD11b + CD45^int^ and ACSA-2, respectively. The samples were centrifuged at 600× *g* for 5 min and the PBS supernatant aspirated, followed by flash freezing in liquid nitrogen and storage at −80 °C prior to sample preparation for mass spectrometry processing.

### 2.8. Mass-Spectrometry-Based Proteomics

Microglia and astrocyte sorted samples were subsequently processed by the iST sample preparation kit (PreOmics, Planegg/Martinsried, Germany) as described previously [61]. Samples were then resuspended in 0.1% formic acid in water and injected on-column via a nanoElute2 (Bruker, Billerica, MA, USA) nanoflow ultra-high performance liquid chromatography (UHPLC) system coupled to a trapped ion mobility spectrometry TIMS-TOF instrument (timsTOF Pro, Bruker, Billerica, MA, USA). A CaptiveSpray ion source with a column oven heated to 50 °C was utilized along with the Aurora Ultimate CSI UHPLC reversed-phase C18 column (25 cm × 75 μm i.d., 1.7 μm C18, IonOpticks, Collingwood VIC 3066, Australia). A separation gradient was applied using mobile phases A (0.1% formic acid in water) and B (0.1% formic acid in acetonitrile). Following column equilibration with 100% A, the total runtime was 120 min, wherein most peptide elution occurred during a 90 min gradient of 2–25% B. The gradient profile also included column cleaning using a rapid increase to high % B (80%), followed by a blank injection to minimize residual carryover. The timsTOF Pro was operated in DIA-PASEF scan mode within an ion mobility range of 0.7–1.40 1/K_0_ [V·s/cm^2^] spanning 250–1425 *m*/*z* (estimated 1.48 s cycle time). A mass width of 25 Da was set, along with one mobility window and no mass or mobility overlap. Collision energy and DIA-PASEF windows were set to 20 eV for a base of 0.60 1/K_0_ [V·s/cm^2^] and 59 eV for a base of 1.60 1/K_0_ [V·s/cm^2^]. Three ions at 622, 922, and 1222 *m*/*z* (Agilent, Santa Clara, CA, USA) were used to calibrate both ion mobility and m/z.

DIA data of each time point and cell type were analyzed separately in DIA-NN (v.1.8.2 beta 22) in library-free mode using an in silico library generated from the Uniprot Mus musculus database (UP000005640, 55,315 entries, downloaded 18 May 2022) with a peptide length range of 7–30, precursor charge range 2–4, precursor *m*/*z* range of 375–1200, and fragment ion *m*/*z* range of 200–1800. Label-free quantification (LFQ) with match-between-runs (MBR) using an FDR cutoff of 1% was applied with the following settings selected: 15.0 mass accuracy and MS1 accuracy, use isotopologues, heuristic protein inference, no shared spectra, single-pass mode neural network classifier, protein inference genes, high precision quantification strategy, RT-dependent cross-run normalization, and smart profiling library generation.

### 2.9. Quantification and Statistical Analysis

Statistical analysis for immunohistochemistry (IHC), lesion volume, and behavioral data was conducted using GraphPad Prism (v9.0.1). For dose–response experiments at 7 days post-injury (dpi), comparisons among treatment groups (sham, TBI + PBS, and TBI + sEVs at various doses) were performed within each age group (3-, 15-, and 20-month-old) using one-way ANOVA followed by Tukey’s post hoc test to identify the optimal dose. At 30 dpi, two-way ANOVA with Tukey’s multiple comparisons test was used to examine the effects of age and sEVs treatment, as well as their interaction, on behavioral outcomes, IHC markers, and lesion volume. To assess longitudinal motor function, EBST scores were analyzed at days 1, 3, and 5 post-treatment for each mouse using repeated measures ANOVA, stratified by age and followed by Tukey’s post hoc analysis. Data are presented as mean ± SEM, with statistical significance set at *p* < 0.05. Corresponding F-values from ANOVA tests are reported.

For proteomic data, statistical analysis was performed on each age group and cell type independently in Perseus (v.2.0.11.0). Specifically, the contaminants were removed from each DIA-NN pg. matrix output file before being uploaded to Perseus. The label-free quantification (LFQ) intensity values were annotated and log_2_ transformed followed by filtering, where 70% of values must be present in total for any given protein to remain. Missing values (up to 30% of any given protein) were replaced through the imputation function using the normal distribution option with a width of 0.3 and a downshift of 1.9 to fit the lower abundance of the Gaussian curve [62]. ANOVA (*p* < 0.05) followed by a post hoc Tukey test (FDR < 0.05) was performed. The proteins that met statistical cutoff were uploaded into Ingenuity Pathway Analysis (IPA), and further comparisons were filtered for *p* < 0.05 (Fisher’s Exact Test) and activation *z*-score cutoff >2.

## 3. Results

### 3.1. hASC-sEV Dose–Response Identifies 20 µg as Optimal for Improving TBI-Induced Motor Deficits in Young and Aged Mice at 7 dpi

**Figure 1:**
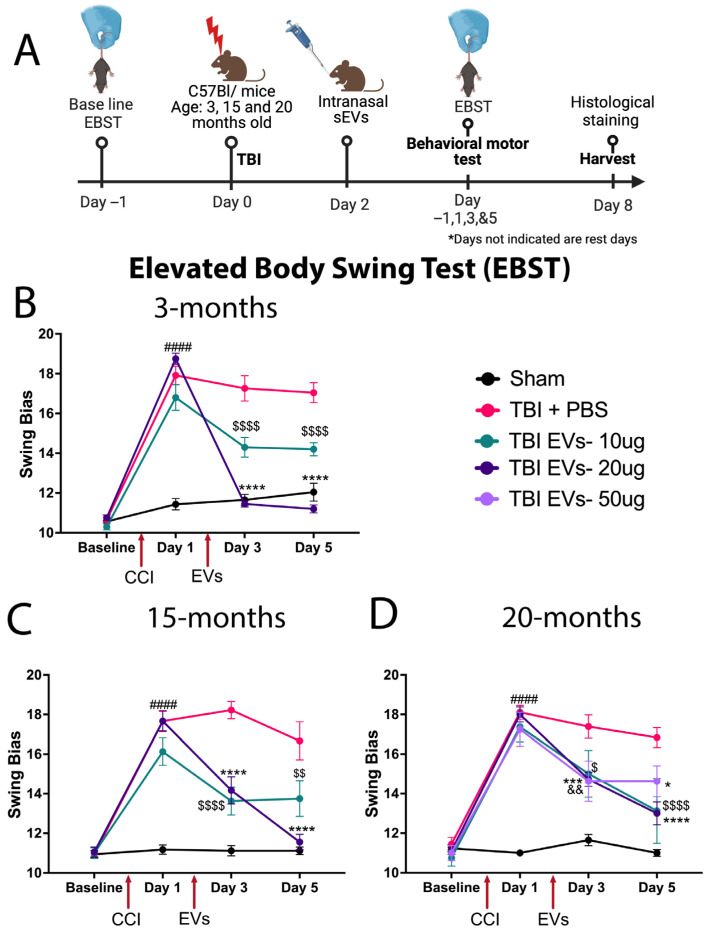
Dose–response study of hASC-EVs to improve motor (EBST) behavior. The results showed that a 20 µg dose was most effective in improving motor deficits in young and aged mice following TBI. (**A**) Schematic timeline of the hASC-derived EV dose response experiment at 7 dpi. (**B**–**D**) Elevated Body Swing Test (EBST), a motor function assessment test showing a 20 µg dose of EVs, the optimal dose that rescued motor function post-TBI compared to 10 µg and 50 µg EVs. Two-way repeated-measure ANOVA shows significant time and dose interaction effects *p* < 0.0001 at (**B**) 3 months (F_(9, 216)_ = 49.93), (**C**) 15 months (F_(9, 171)_ = 16.40), and (**D**) 20 months (F_(12, 192)_ = 12.73). Tukey’s multiple comparison is indicated by (# =TBI + PBS vs. Sham), ($ = TBI + PBS vs. TBI + 10 µg EVs), (* = TBI + PBS vs. TBI + 20 µg EVs), and (& = TBI + PBS vs. TBI + 50 µg EVs). Where (####, $$$$, **** represent *p* < 0.0001, *** *p* < 0.001, $$ or && *p* < 0.01, $ or * *p* < 0.05). Data are presented as mean ± SEM. Experimental groups included 3-month-old mice (Sham, TBI + PBS [N = 23], TBI + 10 µg EVs [N = 10], TBI + 20 µg EVs [N = 20]); 15-month-old mice (Sham, TBI + PBS, TBI + 10 µg EVs [N = 8], TBI + 20 µg EVs [N = 18]); and 20-month-old mice (Sham [N = 17], TBI + PBS, TBI + 10 µg EVs, TBI + 20 µg EVs, and TBI + 50 µg EVs [N = 8]).

EBST was performed prior to CCI to determine the baseline and distribute scores evenly amongst groups. Then EBST was repeated at day 1 post-CCI (before EV treatment), day 3, and day 5 (after EV treatment) as shown in the timeline (Figure 1A). The young and aged mice revealed significant asymmetry in motor activity in all injured mice at day 1 following CCI when compared to sham (Figure 1B–D). Treatment with different doses of EVs at 48 h post-CCI led to significant improvement in EBST behavior compared to TBI + PBS mice at all ages, but there were differences in efficacy. In 3-month-old mice (Figure 1B), both 10 µg and 20 µg doses improved swing bias at day 3 and day 5 (*p* < 0.0001) compared to the TBI + PBS group, though the 20 µg dose showed a larger recovery of swing bias. The 15-month group (Figure 1C) showed a significant decrease in swing bias at 10 µg and 20 µg doses at day 3 post-CCI (*p* < 0.0001), and at day 5 post-CCI, the 20 µg dose showed a continuation of lowering swing bias, which was not observed with the 10 µg dose. The 20-month mice (Figure 1D) were treated with three different doses, 10 µg, 20 µg, and 50 µg, of EVs. At day 3 post-CCI, the EBST bias was reduced compared with sham and TBI + PBS mice (*p* < 0.0257, *p* < 0.0003 and *p* < 0.0058), respectively. At day 5 post-CCI, the doses of 10 µg and 20 µg continued to decrease the swing bias (*p* < 0.0001); however, with the 50 µg dose, the magnitude of the response did not show any further changes.

### 3.2. hASC-sEV Dose–Response Effects Identify 20 µg as Optimal for Reducing the Impact Lesion Volume in Young and Aged Mice at 7 dpi

**Figure 2:**
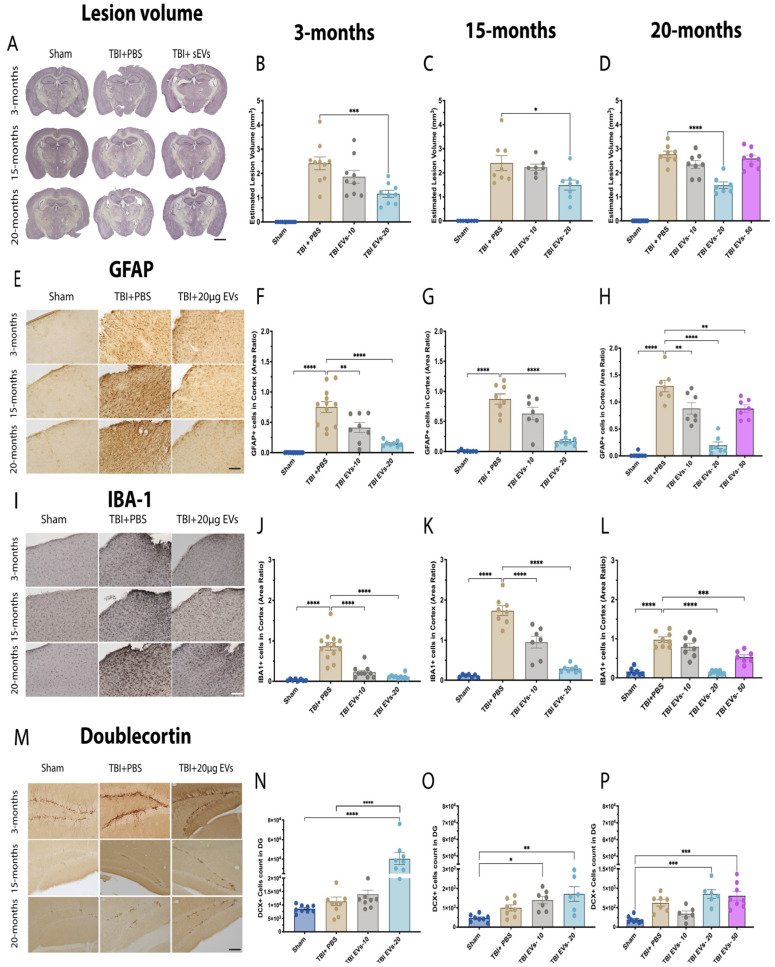
Dose–response of hASC-EVs reduced lesion volume and decreased inflammation markers. The 20 µg EVs treatment post-injury results in decreased lesion volume and decreased area of highly activated microglia and astrocytes in ipsilateral cortex in young and aged mice. (**A**) Stereological assessment of EV treatment in TBI-induced lesion volume at 7 dpi in young and aged TBI mice. Comparison of different EV doses in TBI mice revealed that the 20 µg dose had a significant effect in decreasing lesion volume compared to both the 10 µg and 50 µg doses at (**B**) 3 months, (**C**) 15 months, and (**D**) 20 months. Immunohistochemistry staining was performed for (**E**) GFAP+, and (**I**) IBA-1+ in the ipsilateral cortex of young and aged TBI mice. The 20 µg dose of EVs significantly decreased GFAP+ expression at (**F**) 3 months, (**G**) 15 months, and (**H**) 20 months *p* < 0.0001 and showed the greatest difference between TBI + PBS and TBI + 20 µg EVs compared to the 10 µg and 50 µg doses of EVs post-TBI. The 20 µg dose significantly decreased IBA+ area expression in (**J**) 3-month, (**K**) 15-month, and (**L**) 20-month TBI mice *p* < 0.0001. (**M**) Unbiased stereological assessment of newborn neurons in the dentate gyrus (DG) at 7 dpi in young and aged TBI mice. The 20 µg EVs treatment resulted in a significant increase in DCX+ cell numbers at (**N**) 3 months compared to sham and TBI + PBS (*p* < 0.0001), (**O**) 15 months (*p* = 0.0015), and (**P**) 20 months (*p* = 0.0001) compared to sham. Statistical analysis was carried out by one-way ANOVA followed by Tukey’s post-hoc comparisons. Where *p*-values corresponding to asterisks are as follows: (**** *p* < 0.0001, *** *p* < 0.001, ** *p* < 0.01, and * *p* = 0.05). Data are presented as mean ± SEM. For immunohistochemistry and lesion volume analyses, each experimental group included N = 8–13 animals across different age groups. All immunohistochemistry (IHC) images are presented with a 50 µm scale bar, and lesion volume images are shown with a 500 µm scale bar. Data are presented as mean ± SEM. Experimental groups included N = 8–10.

To further evaluate the effectiveness of 20 µg sEVs, cortical lesion volume in the ipsilateral hemisphere was quantified at 7 dpi using hematoxylin QS staining and the Cavalieri estimator, with images shown in Figure 2A. The quantification in both 3 months and 15 months (Figure 2B,C) showed that TBI mice treated with 20 µg sEVs exhibited a significant reduction in cortical damage compared to TBI + PBS mice (*p* = 0.0022, *p* = 0.0296), respectively. The 20-month (Figure 2D) group was treated with 10 µg, 20 µg, and 50 µg EVs, and only the 20 µg dose demonstrated a significant decrease in lesion volume (*p* < 0.0001) compared to the other doses.

### 3.3. hASC-sEV Dose–Response Analysis Revealed that 20 µg Was Most Effective in Reducing Inflammatory Marker Expression in Both Young and Aged TBI Mice at 7 dpi

Increased glial cell activation has been observed in the aged TBI brain, and it is linked to poorer neurological recovery and increased neurodegeneration when compared to young TBI animals [15,22]. We hypothesize that EVs can mitigate this activation and improve neuronal recovery in young and aged TBI mice. To test this hypothesis, we performed IHC staining analysis for astrocyte markers (GFAP+) and the microglial marker (IBA-1+), as shown in Figure 2. The GFAP images (Figure 2E) show the ipsilateral cortex at 3 months, 15 months, and 20 months. Quantification of GFAP+ (Figure 2F–H) showed that the untreated TBI group exhibited a significant increase in GFAP+ expression compared to the sham group (*p* < 0.0001) at all ages. In TBI mice treated with 10 µg EVs, there was a significant reduction in GFAP+ expression in 3-month and 20-month but not in 15-month mice. The 20 µg dose of EVs showed a reduction in GFAP+ expression (*p* < 0.0001) at all ages. The 50 µg dose of EVs showed less reduction in GFAP+ expression in the 20-month group (*p* = 0.0072). Similar results were observed in the hippocampus, where we observed an increase in GFAP+ at all ages in response to TBI, and the 20 µg dose of EVs was most effective in reducing GFAP+ immunostaining in all age groups (Figure S2A–C) (*p* < 0.05). Staining for IBA+ was also examined with IHC in the ipsilateral cortex at 3 months, 15 months, and 20 months (Figure 2I). The quantification of IBA-1+ staining (Figure 2J–L) showed a significant increase in the TBI + PBS group compared to sham (*p* < 0.0001), which was significantly reduced with 10 µg EVs in 3-month and 15-month but not in 20-month mice. However, a 20 µg dose of EVs demonstrated a significant reduction in IBA-1+ expression within the three age groups (*p* < 0.0001). The 50 µg dose also showed a reduction in IBA-1+ in the 20-month group (*p* < 0.0001). The EVs were demonstrated to significantly reduce IBA-1+ expression in the hippocampus at all three ages following TBI at three different doses (*p* < 0.05) (Figure S2D–F).

### 3.4. hASC-sEV Dose Response for Doublecortin (DCX) Expression in Young and Aged TBI Mice at 7 dpi

To evaluate the impact of sEVs on maturing newborn neurons in young and aged TBI mice, we immuno-stained with anti-DCX and used stereology to quantify the number of DCX+ immunoreactive cells in the dentate gyrus subgranular zone. (Figure 2M) shows DCX+ images for 3-month, 15-month and 20-month mice. Quantification of DCX+ (Figure 2N–P) showed no significant change in the numbers of DCX+ cells with TBI + PBS compared to sham across all age groups. In 3-month-old mice (Figure 2N), TBI + 20 µg EVs significantly increased the number of DCX+ cells compared to either Sham or TBI + PBS (*p* < 0.0001). The DG at 15 months (Figure 2O) showed a significant increase in DCX+ cells in the TBI group treated with 10 µg and 20 µg EVs compared to sham (*p* = 0.0147, *p* = 0.0015, respectively). Although we did observe a significant difference between DCX+ cell counts in TBI + 20 µg EVs and TBI + PBS mice at 3 months, in 15- and 20-month-old mice, there was only a trend to show a difference between TBI + 20 µg EVs and TBI + PBS, but this was not statistically significant. The 20-month mice (Figure 2P) showed a significant increase in DCX+ cell count in TBI treated with 20 µg and 50 µg EVs compared to sham (*p* = 0.0001); however, when comparing TBI treated with EVs to TBI + PBS mice, there was no statistical difference.

### 3.5. hASC-sEV Enhances Cognitive Function in Young and Aged TBI Mice at 30 dpi

**Figure 3:**
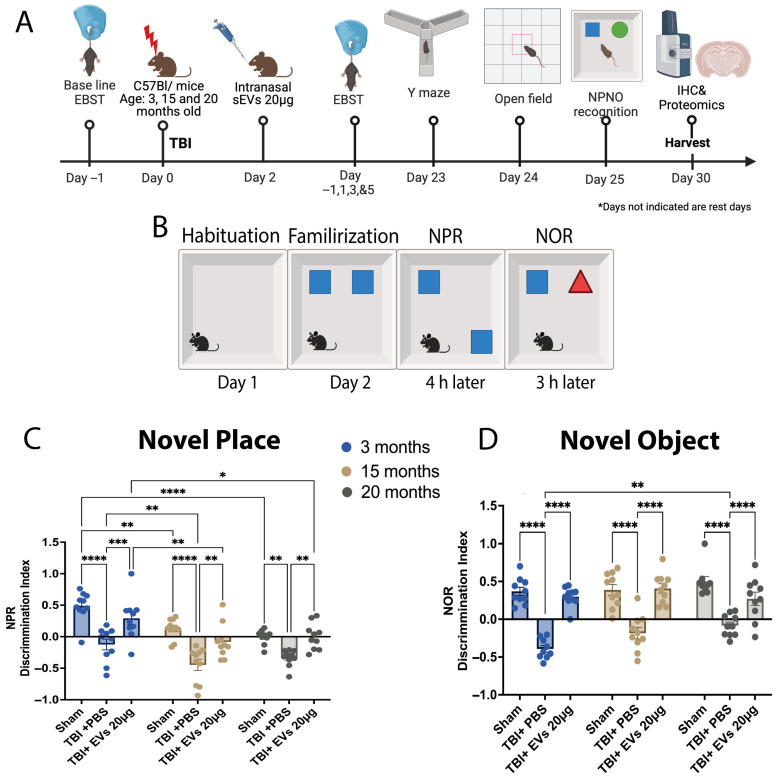
hASC-EVs improved TBI- induced cognitive dysfunction in young and aged mice at 30 dpi. (**A**) Schematic timeline illustrating hASC-derived EV treatment in TBI young and aged mice extended to 30 dpi. (**B**) Schematic the showing novel place (NPR) and novel object recognition (NOR) tests. (**C**) Discrimination index of NPR showing the aging effect in decreasing the recognition of novel place in sham mice as age increases, which significantly decreased following TBI and increased following the 20 µg EV treatment. The post hoc Tukey’s comparisons indicated that age and EV treatment had a significance of *p* < 0.0001 (F_(2, 18)_ = 21.17, F_(2, 18)_ = 37.00, respectively). (**D**) Discrimination index of novel object recognition showing a significant decrease post-TBI and a significant increase with 20 µg EV treatment. The post hoc indicated an EV treatment significance of *p* < 0.0001 and EV and age interaction significance of *p* = 0.0213 (F_(2, 18)_ = 97.06, F_(4, 34)_ = 3.318, respectively). Two-way ANOVA was performed followed by Tukey’s post hoc comparisons. The *p*-values corresponding to asterisks are as follows: * *p* < 0.05, ** *p* < 0.01, *** *p* < 0.001, and **** *p* < 0.0001. Data are presented as mean ± SEM; each experimental group included N = 10 across different age groups.

Once it was determined that the 20 µg dose of sEVs showed the most efficacy, we extended our study to a longer timeline of 30 dpi (Figure 3A) and included cognitive behavior testing. To investigate the animals’ spatial memory and recognition, novel place recognition (NPR) and novel object recognition (NOR) tasks were performed, as well as the Y-maze, test starting at day 23 post-CCI. The open field test (OFT) was performed to identify exploration behavior and as a habituation phase 24 h before NPR and NOR. The OFT showed no significant difference in average speed and distance traveled between ages in different treatment groups (Figure S3A,B). The NPR test was performed, followed by a 3 h interval between the NPR and NOR test (Figure 3B). Discrimination index (DI) of NPR (Figure 3C) was influenced by both age (F_(2, 18)_ = 21.17) and treatment (F_(2, 18)_ = 37.00), (*p* < 0.0001). Post hoc analysis indicated that TBI + PBS mice at all ages resulted in a reduced discrimination index reflecting less time investigating the object in the new place when compared to their sham controls (*p* < 0.05, for each). However, this time significantly increased after EV treatment compared to TBI + PBS mice in an age-dependent manner at 3 months (*p* = 0.0006), 15 months (*p* = 0.0028), and 20-months (*p* = 0.0064). We also observed a significant age effect on performance, as sham at 3 months had a higher DI compared to sham at 15 and 20 months (*p* = 0.0032, *p* < 0.0001, respectively). Similarly, there was a difference between the DI for the TBI + PBS at 3 months compared to TBI + PBS at 15 months (*p* = 0.0091) and TBI + EVs at 3 months compared to TBI + EVs at 15 and 20 months (*p* = 0.0023, *p* = 0.0213, respectively). DI of NOR (Figure 3D) was influenced by treatment (F_(2, 18)_ = 97.06, *p* < 0.0001), but not by age, although there was a significant age and treatment interaction (interaction: F_(4, 34)_ = 3.318, *p* = 0.0213). Post hoc analysis showed that across all age groups, TBI led to a reduced discrimination index compared to age-matched sham controls, indicating less time spent investigating the new object (*p* < 0.0001). However, following EV treatment, this investigation time significantly increased compared to TBI + PBS mice (*p* < 0.0001). Overall, cognitive impairment following TBI in mice was improved in both young and aged mice following the 20 µg EV treatment, showing an age effect in response to EV treatment. The Y-maze test (Figure S3C,D) showed no significant change following TBI in % alternation across age or treatment. There was a significant change (*p* < 0.05) in the number of total arm entries with age in the 20-month group compared with the 3-month group (Figure S3D).

### 3.6. hASC-sEVs Reduce Cortical Brain Injury and Microglial and Astrocyte Activation at 30 dpi

**Figure 4:**
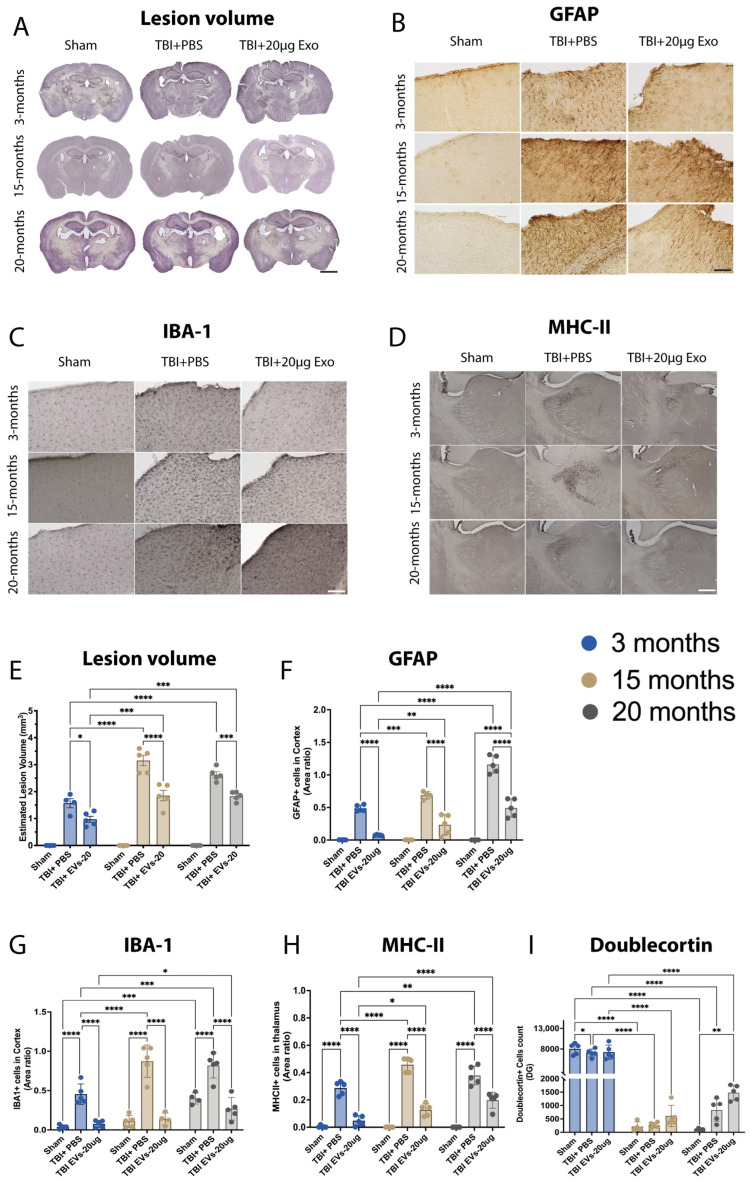
hASC-EVs’ reduced lesion volume and glial cell activation at 30 dpi. (**A**) Images of lesion volume in the ipsilateral cortex. (**E**) Quantification of lesion volume showed age significance (*p* = 0.0002, F_(2, 8)_ = 31.99), 20 µg EV treatment significance (*p* < 0.0001 F_(2, 8)_ = 257.8), and interaction of age and treatment significance (*p* < 0.0001 F_(4, 15)_ = 18.35). (**B**) Images of GFAP+ in the ipsilateral cortex. (**F**) Quantification of GFAP+ immunostaining area revealed age, 20 µg EVs treatment, and interaction of age and treatment significance *p* < 0.0001 (F_(2, 8)_ = 128.0, F_(2, 8)_ = 141.4, and F_(4, 14)_ = 36.95, respectively). (**C**) Image of IBA-1+ in the ipsilateral cortex. (**G**) Quantification of IBA-1+ immunostaining area indicating age significance *p* = 0.0011 (F_(2, 8)_ = 17.84), 20 µg EVs treatment significance *p* < 0.0001 (F_(2, 8)_ = 122.3), and interaction of age and treatment significance *p*= 0.0057 (F_(4, 14)_ = 5.819). (**D**) Image of MHC-II+ in the thalamus. (**H**) Quantification of MHC-II+ immunostaining area revealing age significance *p* = 0.0091 (F_(2, 8)_ = 8.943), 20 µg EVs treatment, and interaction of age and treatment significance *p* < 0.0001 (F_(2, 8)_ = 398.5), (F_(4, 15)_ = 13.82), respectively. (**I**) Quantification of DCX+ cell counts showed no significant increases in TBI + 20 µg EVs compared to TBI + PBS across all age groups; however, there was a significant increase in DCX+ cells of TBI + 20 µg EVs at 3 months compared to TBI + 20 µg EVs 15 and 20 months *p* < 0.0001. Two-way ANOVA was performed followed by Tukey’s multiple comparison. The *p*-values corresponding to asterisks are as follows: (* *p* < 0.05, ** *p* < 0.01, *** *p* < 0.001, and **** *p* < 0.0001). Data are presented as mean ± SEM. For immunohistochemistry and lesion volume analyses, each experimental group included N = 5 animals across different age groups. All immunohistochemistry (IHC) images are presented with a 50 µm scale bar, and lesion volume images are shown with a 500 µm scale bar.

At 30 dpi, brains were harvested, and brain tissues cut and stained with hematoxylin QS staining to quantify lesion volume or immunostained for GFAP+, IBA-1+, MHC-II+, and DCX+. The representative images (Figure 4A) and quantification (Figure 4E) of lesion volume demonstrate that TBI-induced lesion volume was influenced by age, as 3-month mice showed lower lesion volume compared to 15- and 20-month mice (*p* < 0.0001). EVs significantly decreased the lesion volume in all age groups (*p* < 0.05), showing a significant age–treatment interaction F_(4, 15)_ = 18.35, *p* < 0.0001. The representative cortex images (Figure 4B) and quantification (Figure 4F) of GFAP+ revealed higher GFAP+ immunostaining area within 15 and 20 months in TBI + PBS mice compared to 3 months in TBI + PBS mice (*p* = 0.0006, *p* < 0.0001, respectively). A significant reduction in GFAP+ immunostaining area was observed in EV-treated TBI mice when compared to TBI + PBS across all age groups. Similar to what was observed in lesion volume analysis, the EV treatment shows less efficacy within 15- and 20-month TBI-treated compared to 3-month TBI-treated mice to reduce GFAP+ expression (*p* = 0.0013, *p* < 0.0001, respectively). GFAP+ expression was also quantified in the hippocampus (Figure S3E); the results showed a higher expression of GFAP+ area in the TBI + PBS groups in 15- and 20-month compared to 3-month TBI + PBS mice (*p* < 0.05), which was also significantly decreased within EV-treated groups across all age groups when compared to age-matched TBI + PBS (*p* < 0.05). Age also influences the microglia activation in the cortex of TBI + PBS mice, as shown by the representative image of IBA-1+ (Figure 4C) and MHC-II in the thalamus (Figure 4D). The post hoc demonstrated that EVs significantly decreased IBA-1+ (F_(2, 8)_ = 122.3 *p* < 0.0001) in the cortex (Figure 4G) and hippocampus (Figure S3F), as well as the MHC-II in thalamus (F_(2, 8)_ = 398.5 *p* < 0.0001) (Figure 4H); this reduction was age-dependent (interaction: F_(4, 14)_ = 5.819 *p* = 0.0057, and F_(4, 15)_ = 13.82 *p* < 0.0001, respectively). In addition, we immuonostained for DCX+ to assess how aging influences neurogenesis following TBI and the response to EV treatment. The quantification (Figure 4I) showed a significant decrease due to age in the DCX+ cell count (*p* < 0.0001) between young (3-month) and aged (15- and 20-month) mice when comparing across ages for sham, TBI + PBS, or TBI + EVs. However, within each age group, EV treatment did not result in a significant increase when compared to age-matched TBI + PBS mice.

### 3.7. Proteomic Profiling of Microglia and Astrocytes in TBI Mice Following hASC-sEVs at 30 dpi

**Figure 5:**
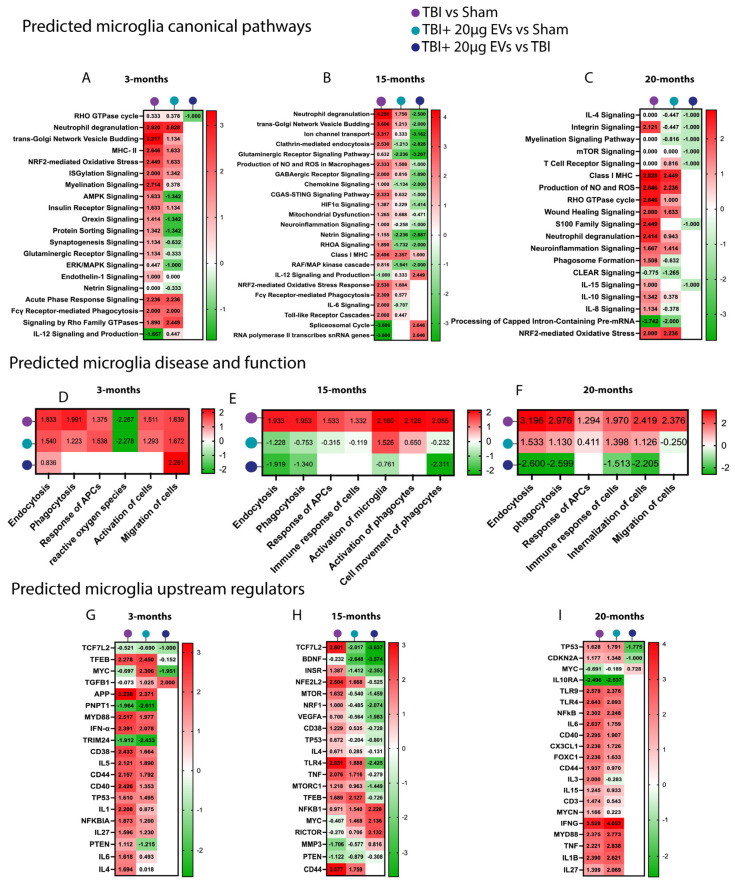
hASC-derived EVs modulated microglia pathways linked to TBI-induced inflammation at 30 dpi. Heat maps annotated by IPA core analysis based on trend and z-score, which represent (**A**–**C**) a comparison analysis of canonical pathways, (**D**–**F**) disease and function pathways, and (**G**–**I**) upstream regulators based on differentially expressed proteins obtained from TBI + PBS/sham, TBI + 20 µg EVs/sham, and TBI + 20 µg EVs/TBI + PBS group comparisons. Three different ages were included: 3 months (**A**,**D**,**G**), 15 months (**B**,**E**,**H**), and 20 months (**C**,**F**,**I**). Red indicates predicted activation (positive z-score), and green indicates predicted inhibition (negative z-score). Pathway activation or inhibition was considered significant with a |z-score| ≥ 2 or ≤−2, respectively. Blank cells represent cases where the *p*-value for overlap did not meet the statistical threshold (*p* < 0.05) and thus where no activation prediction could be made. Each experimental group included N = 5 animals across different age groups.

**Figure 6:**
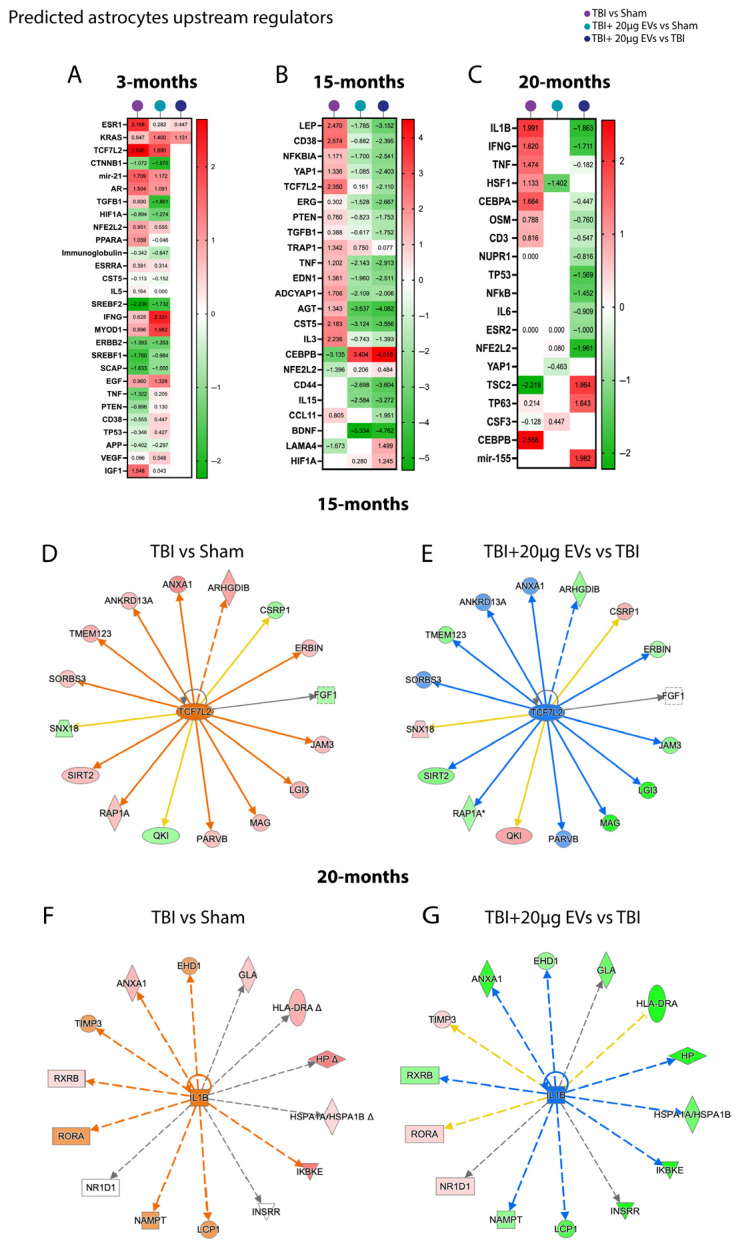
hASC-derived EVs modulated astrocyte upstream regulators linked to TBI-induced inflammation at 30 dpi. EVs shift key upstream regulators (e.g., NFKBIA, TNF, and IL1B) toward the sham level in aged TBI mice at 30 dpi. Heat maps annotated by IPA core analysis based on trend and z-score represent comparison analysis of upstream regulators represented in TBI + PBS/sham, TBI + 20 µg EVs/sham, and TBI + 20 µg EVs/TBI + PBS. (**A**) Three-month mice showed that TBI + PBS/sham had no dramatic change in upstream regulators based on injury effect; similarly TBI + 20 µg EVs/TBI + PBS had no significant changes in upstream regulators based on EV treatment. (**B**) Fifteen months (**C**) and twenty months, where TBI + PBS/sham had upstream regulators that were activated due to the injury, and TBI + 20 µg EVs/sham and TBI + 20 µg EVs/TBI + PBS had upstream regulators that were inhibited due to EVs treatment. Red indicates predicted activation (positive z-score), green indicates predicted inhibition (negative z-score). White boxes without a z-score annotation did not meet the *p*-value cutoff for pathway prediction, and thus no activation prediction could be made. Pathway activation or inhibition was considered significant with a |z-score| ≥ 2 or ≤−2, respectively. IPA analysis shows predicted activation of TCF7L2 at 15 months (**D**) and IL1B at 20 months (**F**) in TBI groups with a z score of 2.350 and 1.991, respectively. The TBI + EVs in the 15-month group (**E**) and the 20-month group (**G**) had predicted inhibition with a z-score of −2.110 and −1.1863, respectively. Each outer node corresponds to an experimentally measured protein, where red signifies up-regulation and green denotes down-regulation. Arrows indicate the predicted relationship to the central node, with blue representing inhibition and orange representing activation. Each experimental group included N = 5 animals across different age groups.

To elucidate the protective mechanisms of EVs against TBI-induced molecular alterations in microglia and astrocytes, mass-spectrometry-based proteomic analysis was performed. Differentially expressed proteins were further analyzed using Ingenuity Pathway Analysis (IPA) to assess predicted pathway activation or inhibition in 3-, 15-, and 20-month-old TBI mice, with or without EVs treatment at 30 days post-injury (N = 5 per treatment group/per age). Comparative analyses of the different experimental groups were performed on isolated microglia and astrocytes from the ipsilateral brain hemisphere. We focused on canonical pathways, downstream functional pathways, and upstream regulators that could contribute to TBI outcomes and are potentially restored by EV treatment. A comparison analysis was performed for the isolated microglia (Figure 5) and astrocytes (Figure 6) based on the trend of the activation z-scores between TBI + PBS/sham, TBI + EVs/sham, and TBI + EVs/TBI + PBS for each age group, with at least one of the comparison groups showing *p* < 0.05 for enrichment significance. This comparison made it possible to identify the trend in predicted pathway activity changes corresponding to aging and EV treatment following TBI.

For the microglial proteome (Figure 5A–C and Supplemental Table S1), we observed canonical pathways linked to inflammation response, where the Z scores showed consistent trends of being increased in TBI and decreased by EVs (i.e., MAPK signaling, MHC class I and II, neutrophil degranulation, neuroinflammation pathway signaling, and proinflammatory interleukins 6 and 15 signaling). This result confirms what we have shown above with IHC, which is further corroborated by studies reporting that TBI activates pro-inflammatory signaling [63]. There was a significant increase in cGAS-STING, an important regulator of innate immune function, following TBI in the 15-month group (Figure 5B), which was decreased with sEV treatment. The results also revealed that excitotoxicity pathways were decreased by sEV treatment. We also observed TBI-induced increases in the production of nitrous oxide (NO) and reactive oxygen species (ROS), mitochondrial dysfunction, and Nrf2-mediated oxidative stress that was decreased by sEVs treatment primarily in the 15-month group. There was also an inhibition of transcription and splicing in response to TBI in the 15- and 20-month groups (Figure 5B,C, respectively), which was reversed by the treatment with EVs. The groups were also sorted based on functional pathways (Figure 5D–F), where microglia showed a trend of predicted activation in the TBI + PBS/sham group in phagocytosis, endocytosis, microglia activation, and immune response of cells, which was inhibited by EV treatment. Within upstream regulator comparisons (Figure 5G–I), we observed a significant change in z-score activation of proteins involved in inflammatory response mediated by TBI, which was decreased in activation with the EV treatment group, e.g., TCF7L2, CD38, CD44, and CD40, IL5, and IL1 at 3 months (Figure 5G) and TNF, TLR4, NFkB, CD44, IL1B, and IFNG at 15 and 20 months (Figure 5H,I, respectively).

For astrocytes, the upstream regulators that were identified using IPA based on astrocytic differentially expressed proteins are shown in (Figure 6A–C and Supplemental Table S2). The 3-month group (Figure 6A) revealed no significant changes at the 30 dpi timepoint. The fifteen-month (Figure 6B) and twenty-month (Figure 6C) groups showed predicted activation of proinflammatory upstream regulators in the TBI + PBS/sham group, such as CD38, NFKB1, TNF, IL1B PTEN, and IFNG, which were inhibited in the TBI + EVs/TBI + PBS group. The 15-month (Figure 6B) TBI + PBS/sham group showed an activation of TCF7L2, the transcriptional regulator of synaptic excitotoxity, which was inhibited with the TBI + EVs/TBI + PBS group. The differentially expressed proteins associated with the predicted activation or inhibition of the TCF7L2 pathway in 15-month-old mice are shown in Figure 6D,E. Similarly, proteins involved in the regulation of IL-1β, a proinflammatory marker, in 20-month-old mice are presented in Figure 6F,G.

## 4. Discussion

It is well established that aging increases the severity of outcomes following TBI [8]. In this manuscript, we extend this to an examination of the impact of age on the response to therapeutic interventions. We demonstrated that there was a therapeutic effect of hASC-sEVs in both young and aged mice following TBI. The sEVs secreted from hASCs are known to carry a diverse array of biological molecules within their complex cargo, including proteins, lipids, mRNAs, miRNAs, and lncRNAs, which provide significant neuroprotection and regeneration after TBI [46]. Our previous work showed that hASC-sEVs delivered intranasally post-CCI were located in a high quantity near the site of injury and observed inside neurons and microglia but not astrocytes. Further, the hASC-sEVs at 10 µg reduced the lesion caused by CCI and lowered neuroinflammation components in young mice [45]. Studies with stem cell approaches have shown reduced therapeutic effectiveness in aged rodents [44]. In this study, we investigated the therapeutic effect of using hASC-derived sEVs delivered intranasally post-CCI in young (3-month) and aged (15- and 20-month) mice. Most studies show therapeutic interventions administered immediately or within a few hours after TBI [64,65,66]. However, this early intervention timing may not be clinically feasible, as many TBI patients with mild TBI do not report immediately for treatment, and some require time for diagnosis before they receive treatment for post-TBI deficits. To address this, we evaluated the therapeutic effect of hASC-sEVs administered at 48 h post-CCI, a translationally relevant time for intervention, and assessed their therapeutic effect at 7 dpi and 30 dpi. We evaluated the therapeutic effects of hASC-sEVs across three age groups of mice, young (3 months), middle-aged (15 months), and aged (20 months), using a dose–response approach. Doses of 10 and 20 µg were tested in the young and middle-aged groups, while an additional 50 µg dose was included for the aged group due to the anticipated diminished responsiveness. The 20 µg dose consistently produced the most favorable outcomes across all age groups, significantly reducing lesion volume and lowering IBA-1 and GFAP expression at the injury site by 7 dpi. Furthermore, treatment with 20 µg hASC-sEVs improved both short-term motor function and long-term cognitive performance.

Motor impairment was evaluated using the Elevated Body Swing Test (EBST), which showed significant motor deficit in the injured young and aged mice following TBI that was attenuated with sEVs treatment. Despite the significant recovery response of motor deficit following EV treatment in both young and aged TBI mice, the aged mice showed slower and reduced recovery following the treatment compared to younger mice. This was consistent with a study of hemorrhage in which aged rats showed greater deficits and slower recovery in forelimb placing and cylinder tests [67]. Our stereological analysis also showed that aged mice have an increased lesion size and reduced response to EVs compared to younger injured mice at 30 dpi. Furthermore, when compared to lesion volume at 7 dpi, the young untreated TBI mice show somewhat lower lesion volume at 30 dpi, demonstrating some natural resolution of the lesion over time, even in the absence of treatment, whereas this is not apparent in either the 15- or 20-month groups.

The combined effects of TBI and aging may have a synergistic deleterious effect on long-term cognitive outcomes. Studies consistently reveal a significant adverse impact of advancing age on functional skills and cognitive abilities following TBI. Studies have shown that aged animals experience a greater impairment in cognitive function compared to younger animals that have been exposed to the same magnitude of TBI [68]. This observation is in line with clinical findings indicating that older patients at the time of injury tend to perform worse on cognitive tasks compared to younger individuals [69]. Similarly, our findings reveal that aged mice had significantly impaired ability to explore novel places and novel objects compared to young mice following TBI. Additionally, we observed that aged sham mice without TBI started with a lower baseline for novel place exploration than young sham mice. Notably, EV treatment significantly improved working memory after TBI across all age groups. It has been suggested that hippocampal neurogenesis plays a role in learning and memory [70,71,72,73,74,75]. In this study, we observed a significant difference in neurogenesis across age groups following TBI and EV treatment. The number of DCX+ cells was increased following TBI with EV treatment in all ages at the 7 dpi time point but not at 30 dpi. At 30 dpi, there was no change with treatment in the young group, suggesting that much of the plasticity had already occurred. However, in the group of 20-month TBI + EVs mice, there continued to be an increase in DCX+ cell number, suggesting a prolonged activation of neurogenesis by the EVs. This difference may help explain the poorer cognitive outcomes associated with older age, potentially resulting from altered plasticity in the aging brain.

It is well established that TBI is associated with secondary inflammation that is responsible for ongoing damage and can last for months [76,77,78,79]. We and others have shown that stem cell approaches, including the use of stem-cell-derived sEVs, can reduce this secondary inflammatory response in young subjects [44,45,80]. In the aging brain, glial cells exhibit a range of functional impairments that contribute to sustained activation, perpetuating a chronic neuroinflammatory state [81]. In this manuscript, we investigated the impact of aging on the response to EV treatment following TBI, with the aim of alleviating the secondary inflammatory response. To examine this, we used both IHC and proteomics approaches. Our IHC analysis revealed an age-related increase in GFAP and IBA-1 expression in the cortex near the lesion site and in the hippocampus, as well as MHC-II in the thalamus, following TBI at both 7 and 30 dpi. EV treatment effectively reversed these elevations across all age groups at 7 dpi; however, by 30 dpi, distinct age-dependent differences in treatment response emerged, indicating an interaction between age and therapeutic efficacy over time. First, the responses of GFAP, IBA-1, and MHC-II to TBI were higher in the older groups. Second, the ability of the EVs to restore GFAP or MHC-II expression levels to baseline sham levels was not observed in the 20-month group. Further, for IBA-1+ expression, the baseline sham value is higher in the aged mice compared to the 3-month mice. Thus, in the older groups, although there is some reduction in GFAP+, IBA-1+, and MHC-II+ in response to the EVs treatment, chronic inflammation remains elevated at the 30 dpi time point. Studies have shown that TBI in the aged brain, when compared with the young brain, resulted in increased microglial proliferation [82] and an associated switch in morphological phenotype from ramified to more hypertrophic or bushy, reflecting a more activated state [23,83]. There was also an increase in MHC-II+ expression [22]. The exacerbated immune response following TBI with aging may correlate to the decreased response to EV treatment post-injury, as aged mice showed a lower, or potentially delayed, ability to resolve inflammation compared to their younger counterparts.

It is widely recognized that both astrocytes and microglia become activated early in aging and following TBI. Microglia can both mitigate and amplify neuroinflammation. An excessive microglial response following TBI, marked by the release of inflammatory mediators, may aggravate brain damage. Recent studies have identified distinct transcriptional profiles in activated microglia in response to various brain insults, including TBI. [84]. We aimed to explore the proteomic changes within microglia and astrocytes following EV treatment in TBI young and aged brains. Our microglial isolation and proteomic analysis provided an indication of the age-dependent microglial response to hASC-derived EVs following TBI. Fifteen- and twenty-month mice had a stronger predicted inhibition of all microglial-relevant functional pathways presented (e.g., endocytosis, phagocytosis, immune response, and microglial activation) in TBI + EVs/TBI + PBS vs. TBI + PBS/sham. In addition, increased predicted inhibition of inflammatory signaling pathways (e.g., neuroinflammation signaling, IL-6, IL-10 pathways, cGAS-STING, and MAPK signaling) in TBI + EVs/TBI + PBS vs. TBI + PBS/Sham was observed. There were fewer significant proteomic changes in the 3-month-old group, which may be indicative of more resolution of changes at this time point (30 dpi). For example, we also showed a smaller lesion volume following injury at 30 dpi in the 3-month-old mice, but not the 15- and 20-month-old mice. The microglia in young TBI mice at 30 dpi may have shown larger downregulation at an earlier time point; for example, in previous publications, it was shown with transcriptomics that at 7 dpi, there was a large down-regulation of pro-inflammatory pathways in young rats treated with hASC EVs [46]. In addition to changes in neuroinflammation signaling, there were also metabolic changes and changes in transcriptional regulation that changed with TBI and with treatment with EVs. The transcriptional changes may be related to the functional changes in the microglia following injury and due to aging, as others observed changes in RNA splicing in different organisms, including mice, as age increased [85]. Transcriptional changes associated with TBI lead to disruption of protein homeostasis as protein synthesis and aggregation are dysregulated with injury [86]. However, more work is needed to directly link changes in RNA polymerase and spliceosome to functional outcomes such as proteostasis in glial cells.

It has been reported that hASC-derived EVs may not enter the cell body of astrocytes, as seen with neurons and microglia [45,87]. When delivered intranasally, we believe EVs might have an indirect impact on astrocytes. This indirect effect could be due to microglia–astrocyte interactions or neuron–astrocyte interactions. Similar to microglia, astrocytes in young TBI mice did not show a significant proteomic change following EVs treatment at 30 dpi, whereas in aged TBI mice, EV treatment led to many changes in astrocytic proteins following TBI. The bioinformatic analysis of differentially expressed proteins in aged astrocytes following EV treatment revealed a significant predicted inhibition of several upstream regulators implicated in neuroinflammation and neurodegeneration (e.g., TNF, IFNG, CD38, NFKB, CCL11, and CD44). Additionally, the predicted inhibition of proinflammatory cytokines (e.g., IL-1B, IL-3, IL-8, and IL-15) as upstream regulators was observed, which was predicted to be activated following TBI. IFNG has been implicated to be associated with changes in T-cells, such as the activation of CD8 T cells; although not examined directly in this study, a clear role for T cells is emerging in the literature on TBI [88]. We also found that changes in TCF7L2, which is associated with the WNT-ß-catenin pathway, could reflect developmental changes in astrocytes following proliferation and/or changes in the blood–brain barrier [89,90].

In conclusion, this study demonstrates that hASC-derived extracellular vesicles (EVs) improve motor and cognitive recovery after TBI in both young and aged mice; however, the therapeutic response is age-dependent, with delayed and attenuated recovery observed in older animals. At 30 dpi, lesion volume and neuroinflammatory markers (IBA1 and GFAP) remained elevated in aged mice compared to their younger counterparts, and proteomic profiling revealed distinct age-specific glial responses, particularly in 15-month-old mice. Despite these promising results, the study has several limitations. First, only male mice were used, limiting the assessment of sex as a biological variable, which is known to influence TBI outcomes and therapeutic responses [91,92]. Future studies should include female subjects to enhance translational relevance. Second, it is possible that age may impact the uptake of sEVs into neuron microglia and other cell types; we have previously examined this in young mice [45], but it is possible that this is altered in the older mice, and further experiments are needed to determine this possibility. In addition, we observed that a higher dose of 50 μg was not effective in the 20-month-old mice, suggesting a U-shaped dose response curve. This would also need further data in mice of different ages to explore this further. Third, in this manuscript, we have not explored the effect of the sEVs in the aged mice without TBI; it is possible that there is some action of sEVs on the baseline pro-inflammatory micro-environment in the aged mice that could be explored and could impact aspects of aging independent of TBI. Fourth, although hASC-sEVs were effective when administered up to 48 h post-injury, delayed access to care remains a clinical challenge. Therefore, future investigations will explore extending the therapeutic window beyond 48 h and assess the efficacy of both single and repeated treatments at later stages post-injury to more accurately reflect real-world clinical scenarios and the chronic progression of TBI.

## Data Availability

The datasets used and/or analyzed during the current study are available from the corresponding author on reasonable request. Proteomic datasets will be uploaded into PRIME and accession #’s (PXD067584) made available.

## Supplementary Materials

The following supporting information can be downloaded at https://www.mdpi.com/article/10.3390/cells14171304/s1, Supplemental Figure S1: Characterization of sEVs: Particle Size, Concentration, and Protein Expression. (A) Nanoparticle tracking analysis results for size distribution and concentration of sEV. Protein detection was performed using automated JESS (ProteinSimple) using antibodies against (B) CD9, (C) ALIX, (D) TSG101 and (E) CD63 to validate sEV markers on the preparation of sEV. In B–E Left panel is the molecular weight ladder, right panel is the JESS bands for each antibody probe, numbers below the panels are protein dilutions run on the JESS capillaries. Supplemental Figure S2: Dose-response of hASC-EVs modified upregulation of GFAP+ and IBA-+ in hippocampus 7 dpi. (A) Immunohistochemistry staining was performed in Hippocampus for GFAP+, and IBA-1+ for young and aged TBI mice. (A–C) The TBI+PBS showed significant increase in GFAP+ area compared to sham matched age group across young and aged mice *p* < 0.0001. (A) 3-months TBI+10 µg and TBI+20 µg EVs showed significant decrease in GFAP+ are (*p* = 0.0015, *p* = 0.0386) respectively compared to TBI+PBS. (B) 15-months TBI+10 µg and TBI+20 µg EVs showed significant decrease in GFAP+ are (*p* = 0.0001, *p* < 0.001) respectively compared to TBI+PBS. (C) 20-months TBI+10 µg, and TBI+20 µg EVs significantly decreased GFAP+ area (*p* < 0.0001) as well as TBI+ 50 µg (*p* = 0.0004) compared to TBI+PBS. (D–F) The TBI+PBS showed significant increase in IBA-1+ area compared to sham matched age group across young and aged mice *p* < 0.0001. (D) 3-months, and (E) 15-months showed TBI+10 µg and TBI+20 µg EVs significant decrease in IBA-1+ are (*p* < 0.0001) compared to TBI+PBS. (F) 20-months TBI+10 µg EVs significantly decreased IBA-1+ area (*p* = 0.0118), as well as TBI+20 µg, and TBI+ 50 µg EVs (*p* < 0.0001) compared to TBI+PBS. Statistical analysis was done by One-Way ANOVA followed by Tukey’s post-hoc comparisons. Data are presented as mean ± SEM, experimental groups included N = 8–10. Supplemental Figure S3: Open field test (OFT) and Immunostaining of GFAP+ and IBA-1+ in Hippocampus at 30 dpi. OFT showed no statistically significant in (A) Average speed and (B) Total distance travelled between TBI+ PBS compared to shame or TBI+ 20 µg EVs in aged matched young (3-months) or aged (15- and 20-months). (C) Y-maze percent alternation showed no significant differences across or within age groups. (D) The number of Y-maze arm entries revealed a significant main effect of age (F(2,18) = 24.2) *p* < 0.05. Post hoc Tukey’s multiple comparisons indicated that arm entries were significantly reduced in Sham, TBI+PBS, and TBI+sEV-treated mice in the 20-month-old group compared to the 3-month-old group. Additional age-related differences were observed between the 3- and 15-month groups in the TBI+PBS condition, and between the 15- and 20-month groups in the TBI+sEV condition. (E) GFAP+ area in Hippocampus revealed an age, EVs treatment and interaction of age and EVs significance *p* < 0.05 (F (2, 8) = 23.74, F (2, 8) = 165.2, and F (4, 14) = 12.85) respectively. Tukey’s post-hoc comparisons showed TBI+ 20 µg EVs significantly decreased (*p* < 0.0001) compared to TBI+ PBS in age matched groups. (F) IBA-1+ area in Hippocampus revealed an age, EVs treatment and interaction of age and EVs treatment significance *p* < 0.05 (F (2, 8) = 36.52, F (2, 8) = 64.37, and F (4, 13) = 8.188) respectively. Tukey’s post-hoc comparisons showed TBI+20 µg EVs significantly decreased in 3-months (*p* = 0.0021), 15- and 20-months (*p* < 0.0001) compared to TBI+PBS. Statistical analysis was was performed using Two-Way ANOVA, and data are presented as mean ± SEM. Sample sizes were N = 10 for the Open Field Test (OFT) and Y-maze, and N = 5 for GFAP and IBA-1 immunohistochemistry. Supplemental Table S1: Quantifiable microglial proteome and differentially expressed proteins (DEPs) across age groups. Microglial proteins were identified and quantified by DIA mass spectrometry in 3-, 15-, and 20-month-old groups. Significant DEPs were determined by one-way ANOVA (*p* < 0.05) followed by Tukey’s HSD test (FDR < 0.05). Highlighted values indicate significant changes for each pairwise comparison. Supplemental Table S2: Quantifiable astrocytic proteome and differentially expressed proteins (DEPs) identified in 3-, 15-, and 20-month groups using DIA mass spectrometry. Significant DEPs were determined by one-way ANOVA (*p* < 0.05) followed by post-hoc Tukey HSD test (FDR < 0.05), highlighted below each average ratio comparison.

## Abbreviations

The following abbreviations are used in this manuscript:

TBI	Traumatic brain injury
CCI	Controlled cortical impact
hASCs	human adipose derived stem cells
sEVs	small extracellular vesicles
CM	conditioned media
dpi	days post injury
OFT	Open field test
EBST	elevated body swing test
NPNO	novel object recognition
SASP	senescence-associated secretory phenotype
TNF-α	tumor necrosis factor alpha
IL-1β	interleukin-1 beta
IHC	Immunohistochemical
PBS	phosphate buffered saline
MHC II	major histocompatibility complex class II
DCX	doublecortin
NO	nitric oxide
PGE	prostaglandins
GFAP	glial fibrillary acidic protein
ANOVA	analysis of variance
IPA	ingenuity pathway analysis
UHPLC	ultra-high performance liquid chromatography
TIMS-TOF	trapped ion mobility spectrometry Time-of-Flight
LFQ	label-free quantification
MBR	match-between-runs
FDR	false discovery rate
IACUC	Institutional Animal Care and Use Committee
