# Supplementary material for: Human Adipose-Stem-Cell-Derived Small Extracellular Vesicles Modulate Behavior and Glial Cells in Young and Aged Mice Following TBI
Source: Cells. 2025 Aug 22;14(17):1304. doi: 10.3390/cells14171304 (PMC12428312; doi:10.3390/cells14171304)
Supplement: Supplementary file 1 [file cells-14-01304-s001.zip › cells-3694485-Supplemental figures.pdf]

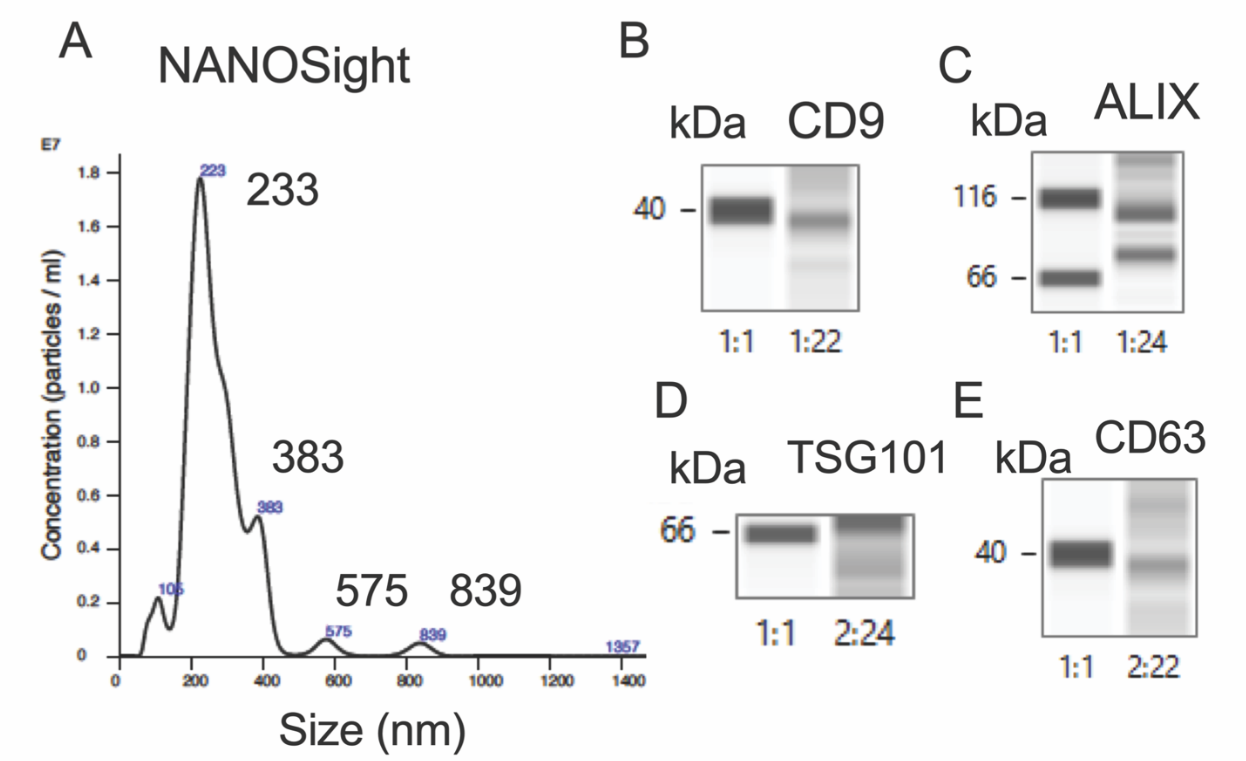

Supplemental Figure S1: Characterization of sEVs: Particle Size, Concentration, and Protein Expression. (A) Nanoparticle tracking analysis results for size distribution and concentration of sEV. Protein detection was performed using automated JESS (ProteinSimple) using antibodies against (B) CD9, (C) ALIX, (D) TSG101 and (E) CD63 to validate sEV markers on the preparation of sEV. In B – E Left panel is the molecular weight ladder, right panel is the JESS bands for each antibody probe, numbers below the panels are protein dilutions run on the JESS capillaries.

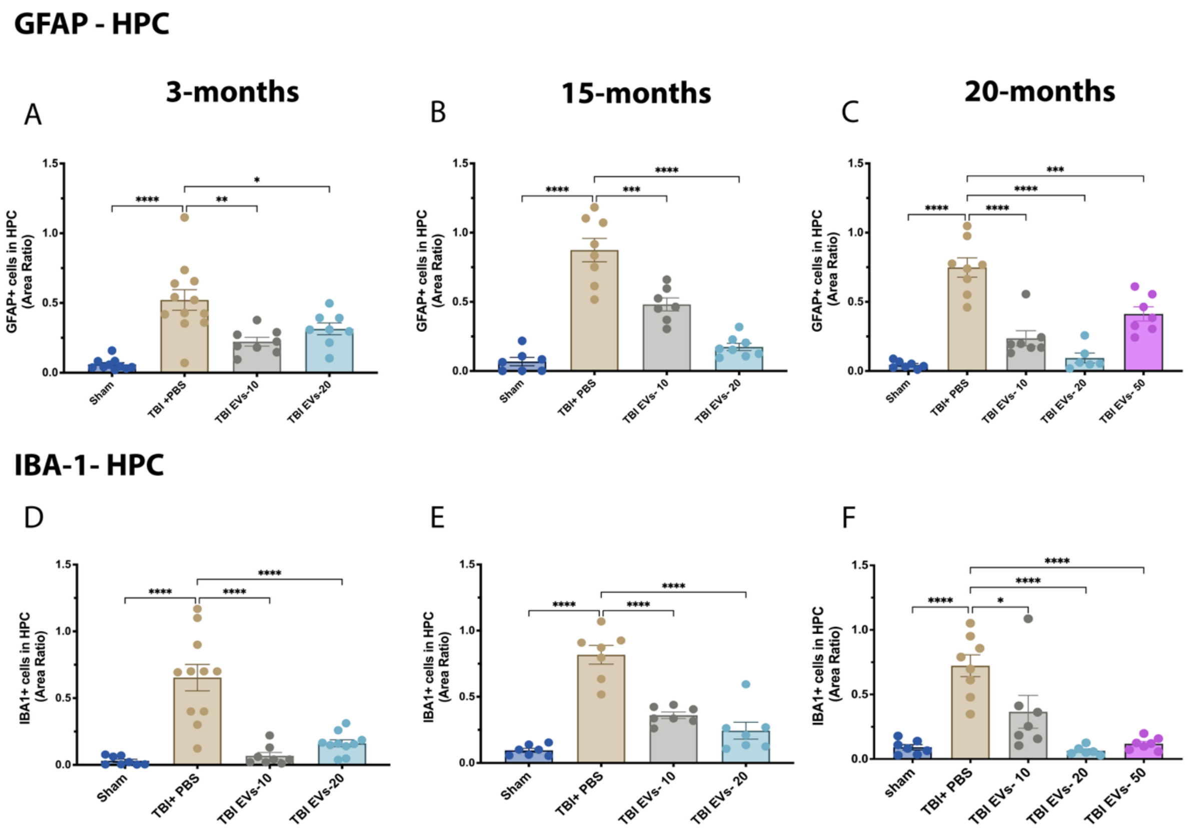

Supplemental Figure S2: Dose-response of hASC- EVs modified upregulation of GFAP+ and IBA-+ in hippocampus 7 dpi. (A) Immunohistochemistry staining was performed in Hippocampus for GFAP+, and IBA-1+ for young and aged TBI mice. (A-C) The TBI+PBS showed significant increase in GFAP+ area compared to sham matched age group across young and aged mice P<0.0001. (A) 3-months TBI+10 µg and TBI+20 µg EVs showed significant decrease in GFAP+ are (P=0.0015, P= 0.0386) respectively compared to TBI+PBS. (B) 15-months TBI+10 µg and TBI+20 µg EVs showed significant decrease in GFAP+ are (P= 0.0001, P< 0.001) respectively compared to TBI+PBS. (C) 20-months TBI+10 µg, and TBI+20 µg EVs significantly decreased GFAP+ area (P<0.0001) as well as TBI+ 50 µg (P= 0.0004)

compared to TBI+PBS. (D-F) The TBI+PBS showed significant increase in IBA-1+ area compared to sham matched age group across young and aged mice $P<0.0001$. (D) 3-months, and (E) 15-months showed TBI+10 μg and TBI+20 μg EVs significant decrease in IBA-1+ are ($P<0.0001$) compared to TBI+PBS. (F) 20-months TBI+10 μg EVs significantly decreased IBA-1+ area ($P= 0.0118$), as well as TBI+20 μg, and TBI+ 50 μg EVs ($P <0.0001$) compared to TBI+PBS. Statistical analysis was done by One-Way ANOVA followed by Tukey's post-hoc comparisons. Data are presented as mean ± SEM, experimental groups included N= 8-10

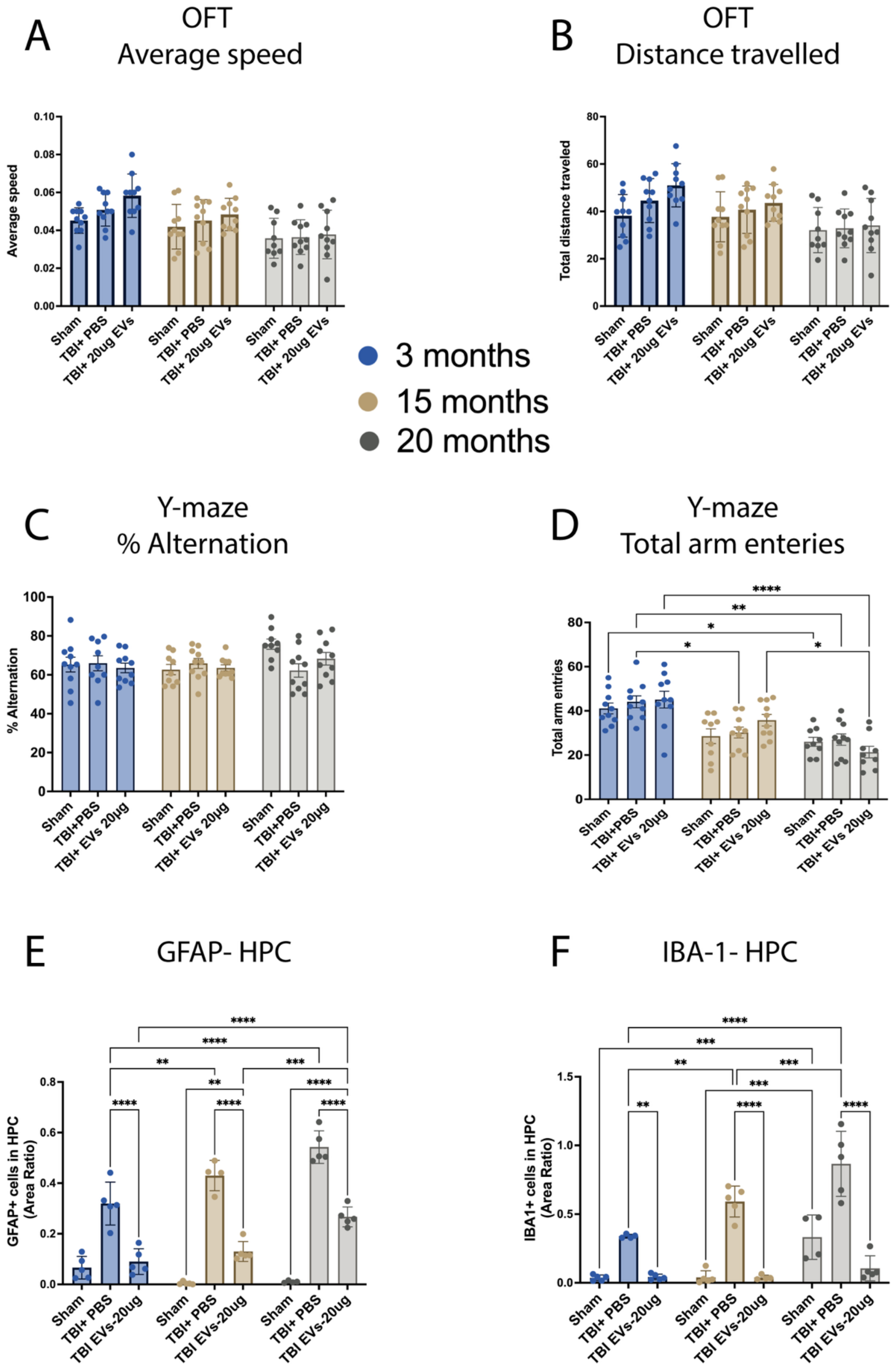

A
OFT
Average speed
Average speed
Sham
TBI+ PBS
TBI+ 20ug EVs
B
OFT
Distance travelled
Total distance traveled
3 months
15 months
20 months
C
Y-maze
% Alternation
% Alternation
TBI+PBS
TBI+ EVs 20µg
D
Y-maze
Total arm enteries
Total arm entries
E
GFAP- HPC
GFAP+ cells in HPC (Area Ratio)
TBI EVs-20ug
F
IBA-1- HPC
IBA1+ cells in HPC (Area Ratio)

Supplemental Figure S3: Open field test (OFT) and Immunostaining of GFAP+ and IBA-1+ in Hippocampus at 30 dpi. OFT showed no statistically significant in (A) Average speed and (B) Total distance travelled between TBI+ PBS compared to shame or TBI+ 20 μg EVs in aged matched young (3-months) or aged (15- and 20-months). (C) Y-maze percent alternation showed no significant differences across or within age groups. (D) The number of Y-maze arm entries revealed a significant main effect of age ($F_{(2,18)} = 24.2$) $P < 0.05$. Post hoc Tukey's multiple comparisons indicated that arm entries were significantly reduced in Sham, TBI+PBS, and TBI+sEV-treated mice in the 20-month-old group compared to the 3-month-old group. Additional age-related differences were observed between the 3- and 15-month groups in the TBI+PBS condition, and between the 15- and 20-month groups in the TBI+sEV condition. (E) GFAP+ area in Hippocampus revealed an age, EVs treatment and interaction of age and EVs significance $p<0.05$ ($F_{(2, 8)} = 23.74$, $F_{(2, 8)} = 165.2$, and $F_{(4, 14)} = 12.85$) respectively. Tukey's post-hoc comparisons showed TBI+ 20 μg EVs significantly decreased ($P <0.0001$) compared to TBI+ PBS in age matched groups. (F) IBA-1+ area in Hippocampus revealed an age, EVs treatment and interaction of age and EVs treatment significance $p<0.05$ ($F_{(2, 8)} = 36.52$, $F_{(2, 8)} = 64.37$, and $F_{(4, 13)} = 8.188$) respectively. Tukey's post-hoc comparisons showed TBI+ 20 μg EVs significantly decreased in 3-months ($P =0.0021$), 15- and 20-months ($P<0.0001$) compared to TBI+ PBS. Statistical analysis was was performed using Two-Way ANOVA, and data are presented as mean ± SEM. Sample sizes were N = 10 for the Open Field Test (OFT) and Y-maze, and N = 5 for GFAP and IBA-1 immunohistochemistry.
